# Development of High Surface Area Organosilicate Nanoparticulate Thin Films for Use in Sensing Hydrophobic Compounds in Sediment and Water

**DOI:** 10.3390/bios14060288

**Published:** 2024-06-03

**Authors:** Sangho Bok, Venumadhav R. Korampally, Jacob K. Stanley, Keshab Gangopadhyay, Shubhra Gangopadhyay, Jeffery A. Steevens

**Affiliations:** 1 Department of Electrical and Computer Engineering, University of Denver, 2155 E Wesley Avenue, Denver, CO 80208, USA; sangho.bok@du.edu; 2 Department of Electrical Engineering, Northern Illinois University, 590 Garden Road, Dekalb, IL 60115, USA; 3 Environmental Laboratory, U.S. Army Engineer Research and Development Center, 3909 Halls Ferry Road, Vicksburg, MS 39180, USA; 4 Department of Electrical Engineering and Computer Science, University of Missouri, 349 Engineering Building West, Columbia, MO 65211, USAgangopadhyays@missouri.edu (S.G.); 5 Columbia Environmental Research Center, U.S. Geological Survey, 4200 New Haven Road, Columbia, MO 65201, USA

**Keywords:** nanosilica, biosensors, environmental sensors, sediments, bioaccumulation, polychlorinated biphenyl

## Abstract

The scope of this study was to apply advances in materials science, specifically the use of organosilicate nanoparticles as a high surface area platform for passive sampling of chemicals or pre-concentration for active sensing in multiple-phase complex environmental media. We have developed a novel nanoporous organosilicate (NPO) film as an extraction phase and proof of concept for application in adsorbing hydrophobic compounds in water and sediment. We characterized the NPO film properties and provided optimization for synthesis and coatings in order to apply the technology in environmental media. NPO films in this study had a very high surface area, up to 1325 m^2^/g due to the high level of mesoporosity in the film. The potential application of the NPO film as a sorbent phase for sensors or passive samplers was evaluated using a model hydrophobic chemical, polychlorinated biphenyls (PCB), in water and sediment. Sorption of PCB to this porous high surface area nanoparticle platform was highly correlated with the bioavailable fraction of PCB measured using whole sediment chemistry, porewater chemistry determined by solid-phase microextraction fiber methods, and the *Lumbriculus variegatus* bioaccumulation bioassay. The surface-modified NPO films in this study were found to highly sorb chemicals with a log octanol-water partition coefficient (K_ow_) greater than four; however, surface modification of these particles would be required for application to other chemicals.

## 1. Introduction

One of the greatest challenges in exposure assessment is to determine the bioavailability of contaminants in multiphasic and complex environmental media such as natural waters, soils, and sediments. Models based on fugacity are used to characterize chemical activity related to various components and types of soils and sediments, each having different contaminant sorption properties and, subsequently, different contributions to exposure and toxicity. Several approaches have been developed to model the bioavailability of both organic and inorganic contaminants, such as the equilibrium partitioning method (EqP) [1], the sediment biotic ligand model (BLM) [2], and acid volatile sulfide simultaneously extracted metals (AVS-SEM) [3]. Over the past couple of decades, advances in contaminant bioavailability assessment have been made through the use of passive sampling devices to collect the bioavailable or free concentration of contaminants in water or porewater in sediment. There is a wide range of passive samplers such as solid-phase microextraction (SPME) fibers, semipermeable membrane devices (SPMD), polyoxymethylene (POM), polar organic chemical integrative samplers (POCIS), peepers, and diffuse gradients in thin films (DGT) [4,5,6,7,8,9]. Porewater concentrations estimated using passive samplers can be used to parameterize models and estimate exposure concentrations to organisms in contact with sediments. Passive samplers have also been demonstrated as valuable tools to predict the bioaccumulation of hydrophobic organic contaminants with a log K_ow_ ranging from 4 to 7 [10].

In general, most passive sampler methods have relied on the adaptation of polymers, membranes, or fibers from other existing applications. Passive samplers function by adsorbing the chemical of interest to the surface of a polymer or absorbing it within the material. For example, the development of SPME techniques has been a major advance toward this end due to their inherent simplicity and the strong relationship they exhibit between chemical adsorption and traditional measures of bioavailability [10].

Recent advances in materials science offer an opportunity to develop novel sorptive materials that can be used for passive sampling of contaminants in water and air, including deployment in soils and sediments, to sample contaminants in porewater and gas phases. Nanotechnology in particular offers a significant potential for application as a technology for measuring porewater contaminants due to their high surface area and sorptive nature [11,12,13]. A high surface area offers a greater surface for contaminants to bind and, therefore, increases the capacity of the sampler [14,15]. Furthermore, many of these nanoscale particles can be modified or functionalized with chemical groups to bind with a specific chemical [16,17,18] or impart a functional property such as increased fluorescence after the analyte has come into contact with the particles [19,20,21].

The use of polymeric coatings such as thin film microextraction and mesoporous techniques have been developed for extraction and detection of contaminants [22,23,24]. Nanoporous extraction phases are advantageous due to their large specific surface areas [25,26]. Commonly pursued techniques for the synthesis of nanoporous extraction materials include “sol-gel” approaches that employ surfactants and nanopore formation additives [27,28]. Sol-gel processes involve a colloidal solution (sol) of nanoparticles that gel into an organized structure guided by the template set up by a surfactant, followed by controlled evaporation of the solvent to form the solid porous network. Nanoporosity is introduced by forming a nanocomposite film comprising a matrix material and a thermally labile species (porogen) followed by a high-temperature heating step. Removal of the porogen leaves behind nanopores in the matrix material. The porosities of these films thus depend on the initial porogen loading and are constant for a specific loading percent. Both ordered and random pore structures can be formed following this approach by the careful choice of the porogen, templating agent, and the chemistry of the matrix material. The nanoporous films thus formed have random porosity with large pores and are highly susceptible to moisture absorption unless post-treatment of the films is performed to render them hydrophobic.

The preparation of nanoporous structures requires highly controlled reaction parameters and curing processes to avoid pore collapse. These films, with their rigid structures, regularly suffer from large residual thermal stresses, which may initiate cracking and buckling, especially when thick films are desired [29,30]. An alternative method of preparing nanoporous films is through the deposition of nanoparticles, wherein the porosity arises from the volume between individual nanoparticles in the film. For example, nanoporous films can be fabricated by deposition of gas-phase silicon nanoparticles followed by oxidation [31]. Such inter-connected porous morphologies are attractive as extractors (samplers) of chemical contaminants in the aquatic environment because the entire volume of the phase is wetted, thus maximizing the amount of contaminant adsorption. For the extraction of hydrophobic contaminants, additional surface functionalization with hydrophobic moieties during the sol-gel process is necessary to improve the adsorption. Other options such as vapor deposition processes are not readily adaptable to coating three-dimensional shapes (e.g., a glass fiber).

We previously have reported a novel technique to rapidly and controllably synthesize highly porous, large surface area nanoporous organosilicate (NPO) thin films from colloidal dispersions of polymethylsilsesquioxane-coated (PMSSQ) nanoparticles [32]. PMSSQ has been selected as a silica nanoparticle to create a hydrophobic surface that can sorb hydrophobic chemicals. Furthermore, the hydrophobic PMSSQ surface is less susceptible to degradation and fouling in water because of minimal adhesion by bacteria and cells [33]. In this solution-processed deposition technique, the entropy of PMSSQ nanoparticles, which in turn is a function of the curing/calcination temperature, largely determines the final film properties. Thus, porous films with tunable pore-volume fractions may be readily deposited from the same precursor solution but processed using different curing or calcination temperatures.

The purpose of this study was to examine the potential use of nanoparticle-based films as a novel extraction platform for future sorption-based sampler development. Because this is a novel application, some optimization of the film fabrication was required. Films were exposed to trace concentrations of polychlorinated biphenyl (PCB) congener 153 (2,2′,4,4′,5,5′-hexachlorobiphenyl) in aqueous solutions to evaluate uptake potential in a simple system and guide the fabrication conditions. We chose PCB because it is a class of chemicals that are frequently found in the environment and a contaminant of concern at numerous cleanup sites [34]. Lastly, the potential application of NPO films was demonstrated, leveraging an assessment of PCB field-collected sediment by Ingersoll [35] with known PCB contamination, and the results were compared to the bioaccumulation of PCB in a standard test organism, *Lumbriculus variegatus*, with the chemical concentration in whole sediment and the concentration of PCB in porewater determined using SPME.

## 2. Materials and Methods

### 2.1. Nanoporous Organosilicate Thin Film Fabrication

Commercially available PMSSQ (GR650F, Techneglas, Inc., Perrysburg, OH, USA) was used for the preparation of PMMSQ nanoparticles. Propylene glycol methyl ether acetate (PGMEA 98%, Sigma-Aldrich, Milwaukee, WI, USA) and polypropylene glycol (PPG, average molecular weight (*M*_n_) of 425, Sigma-Aldrich) were used as received. The preparation of precursor solutions follows the procedures employed by Korampally [32]. Briefly, 50% (*w*/*w*) solutions of PMSSQ and PPG in PGMEA were prepared separately and sonicated to ensure complete dissolution of the respective solutes. The PPG was used as an initial dispersant for the PMSSQ nanoparticles. The PGMEA was used as a solvent for the PMSSQ and PPG. These individual stock solutions were then mixed together and sonicated to achieve a homogenous mixture with a final PMSSQ:PPG:PGMEA composition of 1:1:2 (*w*/*w*/*w*). All films were deposited from this freshly prepared precursor solution.

Films were deposited onto 2 cm × 2 cm hydrogen-passivated silicon substrates by spin coating at 3000 rpm for 30 s followed by immediate heating on a preheated hotplate. Hydrogen passivation was achieved by briefly dipping the silicon substrates in a 1:10 HF:DI (*V*/*V*) for 10 s immediately prior to the film deposition. Test samples were cured at temperatures ranging from 250 to 550 °C for 5 min after which the films were removed from the hotplate and allowed to cool down to room temperature.

Optical characterization of the films was performed using variable angle spectroscopic ellipsometry (VASE^TM^, J.A Wollam, Inc., Lincoln, NE, USA) to obtain their thickness and refractive index/pore volume fractions (measured at 630 nm wavelength) by applying the Bruggemman effective medium approximation model [36]. For surface area measurement, nanoparticulate powder, obtained by carefully scraping the film from the substrate, was degassed at room temperature for twelve hours and then nitrogen adsorption–desorption isotherms were measured using a Quantachrome Autosorb-1 automated gas sorption system. The surface areas of the samples were computed using the Brunauer, Emmett, and Teller (BET) method.

### 2.2. Preparation and Analysis of NPO Films

The narrow pore diameter and hydrophobic character of the surface limit the wetting of the surface and infiltration of water into the pores via capillary action. To overcome this, the pores of the NPO matrix were filled with water prior to use as a sampler. A two-step procedure was followed prior to deployment of the films in the test media that employed a methanol exchange step that was performed to facilitate the infiltration (wetting) of the aqueous test solution into the pores. Briefly, the methanol-assisted infiltration was completed by covering the surface of the film with methanol, pouring off the excess, and then immediately placing the film into a 250 mL bowl containing 100 mL water for 30 min. This bowl was agitated slightly for this time period using a rotary shaker table. The NPO films were then stored in water until use.

### 2.3. Assessment of NPO Film PCB153 Adsorption Capacity

Polychlorinated biphenyl (PCB) congener 153 (2,2′,4,4′,5,5′-hexachlorbiphenyl) (CAS# 035065-27-1, ULTRA Scientific, North Kingstown, RI, USA) was used as a model hydrophobic organic contaminant. This specific congener was chosen to represent a mid-molecular weight PCB congener that is likely to be found in contaminated sediments. PCB153 was added to water using semipermeable membrane devices (SPMD), passive dosing devices made of low-density polyethylene (LDPE) tubing containing the neutral lipid triolein. Although SPMDs are most commonly used as sampling devices, they can also be used as passive dosing devices by placing chemicals into the SPMD and allowing the chemicals to partition out into the surrounding water [37]. In this case, we chose to use the passive dosing approach to provide a stable concentration of PCB 153 in the exposure system and prevent depletion of the water by the sampling device.

The LDPE tubing, obtained from Environmental Sampling Technologies (St. Joseph, MO, USA), was 2.5 cm wide with a wall thickness of 100 µm. Each SPMD was made of a 10 cm length of LDPE tubing and contained 0.1 g of triolein (glyceryl trioleate, Sigma-Aldrich Chemical Company, St. Louis, MO, USA) spiked with 1 mg of PCB153. PCB153 was dissolved in 2 mL of hexane for loading into the SPMDs. The hexane-containing PCBs were then layered on top of a known mass of triolein. The hexane was then evaporated using a stream of high-purity nitrogen gas, allowing the PCBs to partition into the triolein. The triolein was then vortexed at high speed for three minutes to ensure homogeneity. The triolein PCB mixture (0.1 g) was then loaded into a 10 cm section of the LDPE tubing that had one end sealed with an impulse heat sealer (model 216x, Fisher Container Corp., Buffalo Grove, IL, USA) using a glass pipette. The mixture was distributed evenly throughout the LDPE tubing by pressing the rounded edge of the glass pipette against the outside of the tubing, and the other end of the tubing was sealed using the heat sealer. A small loop was created at the end of the LDPE tubing using the heat sealer, and a Teflon-coated stir bar was inserted in this loop to act as a weight to prevent the SPMD from floating.

SPMDs with Teflon-coated stir bars were added into a beaker containing 2 L of dechlorinated laboratory water (hardness 110–120 mg/L as CaCO_3_, dechlorinated via carbon filtration). All beakers were silanized using 5% dimethyldichlorosilane in toluene (Sylon CT, CAS Number: 75-78-5, Sigma-Aldrich Chemical Company, St. Louis, MO, USA) to minimize adsorption of PCB153 to glassware. Trickle flow aeration (approximately 1 bubble per s) was performed to ensure continuous movement of water within the beakers, and the beakers were loosely covered with plastic film to prevent water evaporation. The PCB153 spiked SPMDs were placed in the beakers of water for 7 d prior to the addition of NPO film.

Two controlled experiments with PCB153 in water were conducted to optimize the NPO film fabrication for maximum adsorptive capacity for PCB153 and to determine the rate of PCB153 adsorption to the NPO film. The temperature at which the NPO films are cured ranges from 250 to 550 °C and directly affects their porosity and, therefore, the accessible sorptive surface to which the target chemical can bind. To determine the optimal temperature for fabrication NPO films were cured at 250, 350, 450, and 550 °C. The thickness and surface area of the resulting films were determined as described above. NPO films in triplicate were immersed in 2 L of water containing a concentration of 0.006 ± 0.003 ng/mL PCB153 for seven days at 23 °C. The 550 °C fabrication temperature NPO film had the highest amount of PCB153 sorbed and thus was selected. In a second experiment, the adsorption of PCB153 on the NPO films cured at 550 °C was determined over a 7-day exposure period. The NPO films were placed in individual replicate 2 L beakers using the passive dosing system described above. Three replicate NPO films were sampled at 12, 24, 72, 120, and 168 h. A 1 L water sample was collected from each replicate 2 L beaker at the time the NPO films were sampled for chemical analysis.

The NPO films were removed from the water and PCB153 was extracted by submerging the film in 3 mL of hexane in a 50 mL beaker for 30 min. An additional 2 mL of hexane was used to rinse the NPO film after removal from the beaker and the beaker itself after contents were poured into a 20 mL glass scintillation vial where each sample was stored. Each vial was tightly capped and individually wrapped around the cap and neck of the vial in self-sealing plastic laboratory film to a tight seal. Vials were placed in a 4 °C refrigerator until chemical analysis. After extraction of the NPO films, the surface area of the NPO films was measured using Image-Pro Plus 7.0 (Media Cybernetics, Bethesda, MD, USA) image analysis software on digital photographs of each film.

The concentration of PCB in the NPO film hexane extracts and water was determined using gas chromatography with an electron capture detector following USEPA Method 8082A [38]. The reporting limit for PCB in water samples from the exposure chamber was 0.002 ng/mL. Recovery of PCB153 was 80.7% for matrix blank water samples and 81.2% for surrogate recovery standard 2,4,5,6-tetrachloro-m-xylene solution (TMX), which was added to each sample before extraction. The response of the instrumental internal standard (IS) PCB-209, which is added to the final extract just prior to GC analysis, was 88.7%.

### 2.4. Application of the NPO Film to Assess PCB in Sediment

To determine the potential utility of the NPO film platform to estimate free PCBs in porewater, we compared the uptake of the NPO film to measures of PCB bioavailability as part of a site evaluation at Anniston, AL, USA [35]. The Anniston site was located in northern Alabama and includes a portion of Choccolocco Creek that was contaminated with PCBs. To assess the bioavailability of the PCB and potential bioaccumulation, multiple lines of evidence were used, including modeling PCB availability using equilibrium partitioning, measurement of porewater PCB using SPME fibers, and direct measurement of PCB bioaccumulation using the oligochaete, *Lumbriculus variegatus*. A total of 14 sediments from this study were used to evaluate the uptake of PCB onto the NPO films and then compared to the other measures described in the Anniston PCB assessment.

The NPO films were placed in Anniston sediments for 28 days (1 replicate per sediment) using the same methods used to deploy SPME fibers described by Steevens [39]. Because PCBs are stable in sediments, we expected very little difference in PCB concentrations. Briefly, NPO films fabricated as described above and cured at 550 °C were deployed in 2 L beakers containing 600 mL of sediment and 1400 mL of overlying water. Each NPO film was placed into a 100 µm stainless-steel mesh envelope to protect it from physical damage when placed in the sediment. Exposure beakers were treated the same as bioaccumulation exposure chambers and contained 2.66 g of *Lumbriculus variegatus* and received water changes three times weekly. After 28 days, the NPO films were removed from the sediment and rinsed with ultrapure water. The NPO film was removed from the stainless-steel envelope and rinsed with ultrapure water and PCB was extracted following the same procedures described above for the controlled experiments with PCB153 in water.

### 2.5. Statistical Analysis

Data are made publicly available through a data release [40]. All statistical analyses including analysis of variance and linear regression were performed using GraphPad Prism version 7.00 for Windows, GraphPad Software, La Jolla, CA, USA. Statistical significance was assessed at α = 0.05. The relative performance of NPO films created using different temperatures was assessed using a one-way analysis of variance.

## 3. Results and Discussion

### 3.1. NPO Film Characteristics and Optimization

Fabrication of the NPO film resulted in a porous high surface area coating with adequate porosity for the aqueous sample to reach the interior surfaces. Previous work in our laboratory showed the size of the organosilica nanoparticles on the film to average 5 nm in diameter with a high surface area [32,41,42]. Optimization of the curing temperature to produce the highest surface area and porosity was determined experimentally. The thickness of the film declined from 250 to 550 °C; however, the volume-normalized PCB153 sorption was highest with 550 °C curing (Table 1). Therefore, all the experiments were conducted using the 550 °C cured films.

**Figure 1:**
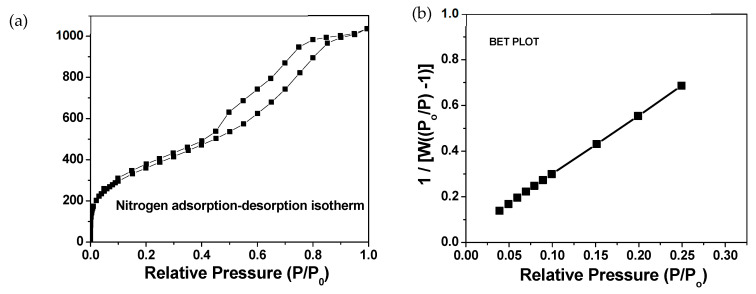
(**a**) Nitrogen adsorption–desorption isotherm measured for nanoporous organosilicate (NPO) films and (**b**) calculation of Brunauer–Emmett–Teller (BET) surface area by 10 data points in low relative pressure.

The average refractive index of 17 NPO films prepared at 550 °C and used for the PCB sorption study was 1.16 ± 0.01, and the average thickness was 638 ± 88 nm. The BET analysis (Figure 1a) revealed that the surface area of nanoparticulate porous PMSSQ film is 1325 m^2^/g. The contribution of the microporosity (porosity less than 2 nm as defined by the International Union of Pure and Applied Chemistry (IUPAC)) to the total surface area estimated from a t-plot analysis is 42 m^2^/g. The isotherm obtained for these films shown in Figure 1b is classified as Type IV, as normally observed in typical mesoporous samples (pore size of 2–50 nm as defined by IUPAC). However, it is seen that there is a sharp increase in the volume of nitrogen adsorbed at relative pressures (P/P_o_) in the range of 10^−7^ to 10^−4^. Therefore, it is expected that these samples exhibit both microporosity and mesoporosity. While some microporosity is observed in the NPO film, the high surface area of these samples is attributed to the large number of pores created by the removal of PPG, voids between the nanoparticles, and the extremely small size of these nanoparticles with a narrow size distribution.

**Figure 2:**
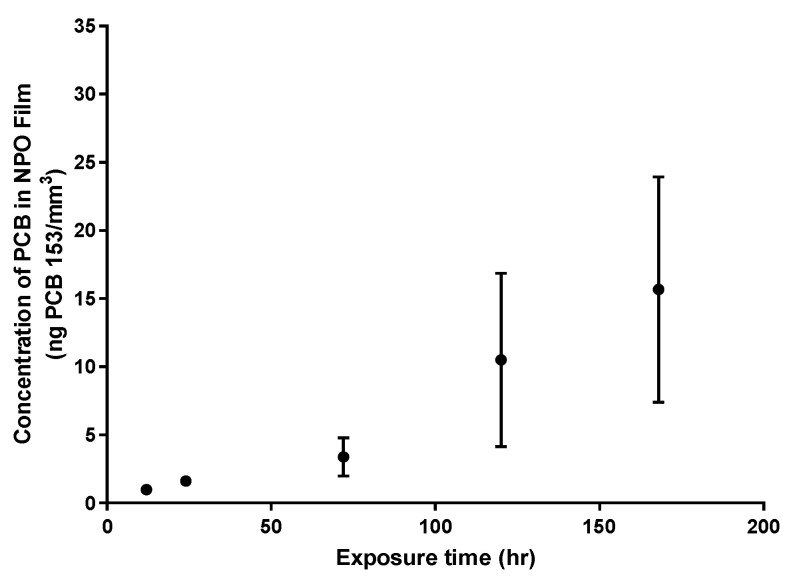
Sorption of polychlorinated biphenyl congener (PCB) 153 to the nanoporous organosilicate (NPO) thin film in water. The concentration of PCB 153 in water during the sorption experiment was 0.07 ± 0.9 ng/mL. Points represent an average of three samples and the standard error of the mean. Error bars at 12 and 24 h time points are less than 0.27 ng/mm^3^.

The model chemical PCB153 was used to guide the fabrication of a sorptive film for PCBs in water. The 550 °C NPO films exposed for 7 days to PCB153 in water have demonstrated the sorption of PCB153 to the NPO surface (Figure 2). Optimally, uptake kinetics could be calculated as part of this experiment if the PCB water concentration was constant. However, it appeared the sorption rate of the PCB to the NPO film might exceed the rate of PCB depuration from the SPMD dosing system. The initial equilibrated PCB153 concentration in the water prior to placing the film sampler was rapidly taken up by the NPO film. The average concentration of PCB153 over the duration of the study was 0.07 ± 0.09 ng/mL in the water. The high variability associated with the 5-day time point was apparently due to an exceptionally high measured water concentration of PCB153 (0.386 ng/mL) in one of the three replicates from this time point and not due to variability in the NPO films themselves. The other two replicates assessed at the 5-day time point had water concentrations of PCB153 of 0.0149 and 0.0210 ng/mL. We suspect difficulty in analyzing these low levels of PCB153 in water may have contributed to the highly variable measured concentrations in the water.

### 3.2. Application of NPO Film Platform to Assess PCB in Sediment

**Figure 3:**
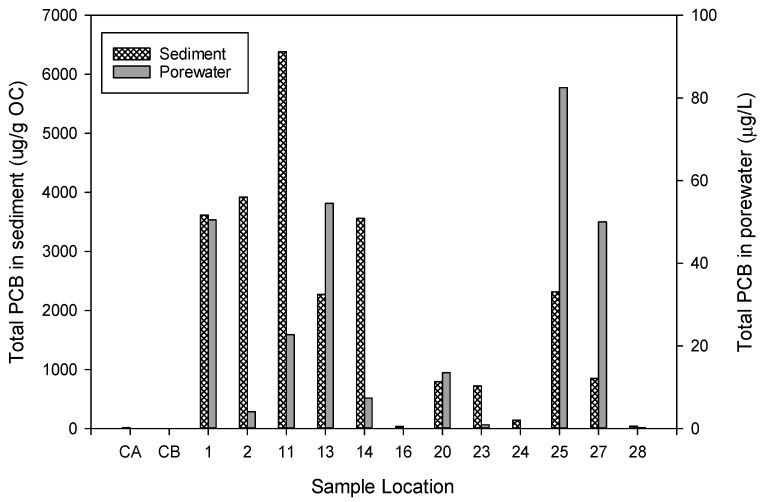
The concentration of polychlorinated biphenyls (PCB) in sediment and porewater, measured by solid-phase microextraction fiber from the Anniston AL site assessment described by Ingersoll et al. [35] Sites 1 through 28 are samples from Choccolocco Creek; AL and CA and CB are the control samples for batch A and batch B.

The utility of the NPO film for use as a sediment porewater sampler was demonstrated in the PCB-contaminated Choccolocco Creek. The total PCBs, reported as a sum of PCB homologs, in sediment ranged from 4.9 to 6380 µg/g organic carbon and in porewater up to 82.5 µg/L (Figure 3). Data from laboratory testing of NPO films with Choccolocco Creek sediment demonstrate that it functions as a passive sampler platform to assess a hydrophobic chemical in whole sediment.

NPO films continued to collect PCBs from the sediment porewater over the 28-day exposure. The concentration of PCB collected by the NPO film, normalized to the pore volume of the film, ranged from 91 to 2736 µg/mm^3^ and correlated to the concentration of PCB in the sediment. It appears the hydrophobic nature of the PMSSQ surface coating on the silica nanoparticles provided an adequate substrate for the sorption of PCB from the sediment and porewater in this sediment system. None of the films, protected by the stainless-steel housing, were damaged during placement in the sediment system and were easily recovered and extracted. Hydrophobic surfaces, such as the NPO film, typically have a lower potential for adhesion-based fouling than many hydrophilic surfaces.

**Figure 4:**
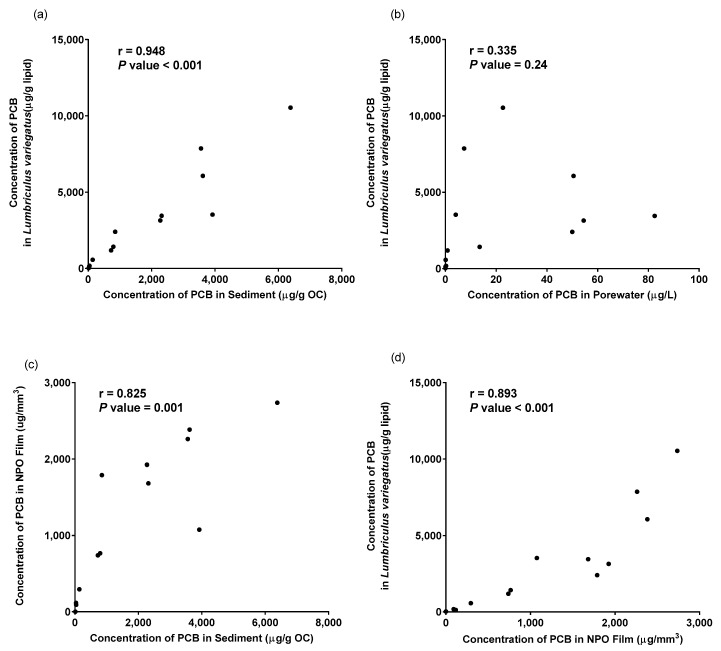
Correlation of total polychlorinated biphenyls (PCB) in sediment (**a**,**c**), porewater (**b**), *Lumbriculus variegatus* (**a**,**b**,**d**)*,* and NPO thin films (**c**,**d**). Pearson correlation coefficients are indicated on the upper left of each figure.

Ingersoll [35] reported that the PCB concentrations in sediment porewater, as assessed by porewater estimated using SPME and bioaccumulation by oligochaetes, followed classic equilibrium partitioning. PCB normalized to organic carbon in sediment, was significantly correlated with lipid-normalized PCB in the tissue of the oligochaete (Figure 4a). However, PCB homologs assessed using the SPME fiber to estimate porewater PCB concentrations were relatively weak as shown in Figure 4b. Because the NPO films were deployed in the same sediments using the same exposure conditions, the results of the sediment assessment can be directly compared to the uptake of PCB homologs by the NPO films. There was a significant correlation (r = 0.68, *p*-value = 0.001) between organic carbon-normalized PCB in sediment and PCB in the NPO film (Figure 4c). This suggests the NPO film provides a measure of the bioavailable fraction of PCB in these sediments. Furthermore, a significant relationship (r = 0.80) was observed between the PCB in the NPO film and the total PCB in the oligochaete (Figure 4d). This correlation was similar to the correlation using organic carbon-normalized sediment concentrations.

**Figure 5:**
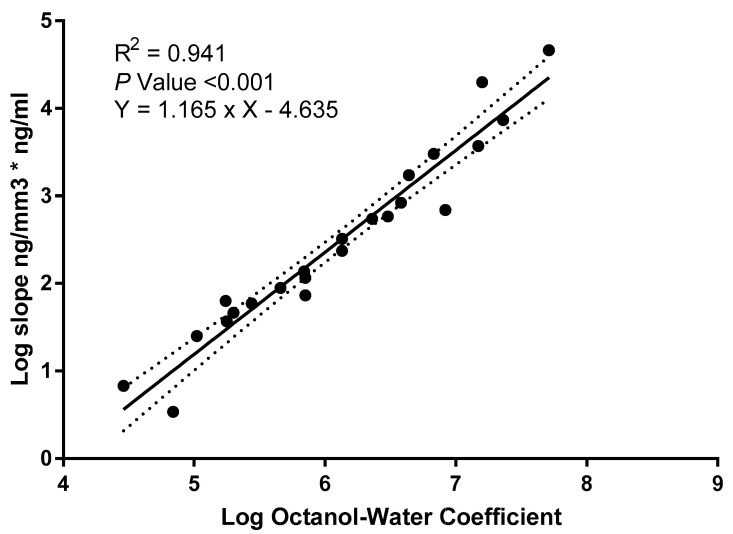
Regression (with 95% confidence bands) of the log octanol-water partition coefficient (K_ow_) versus the concentration of polychlorinated biphenyl (PCB) in nanoporous organosilicate (NPO) sampler normalized to the concentration of PCB in the sampled sediment porewater.

Evaluation of the NPO film using total PCB provides limited information regarding the sorption of individual PCBs having different hydrophobicities. Therefore, we further evaluated the differential sorption of PCB congeners in porewater estimated using the SPME compared to the concentration of PCB in the NPO film. Using 23 different congeners, we calculated a regression slope between PCB in porewater estimated using SPME versus the concentration of PCB in the NPO film. PCB congeners that were not detected in the sediment samples were excluded from subsequent analysis. The log of the NPO film normalized to the porewater concentration to porewater concentration was plotted against the octanol-water partition coefficient (K_ow_) [43] for each respective congener as shown in Figure 5. This highly significant correlation (*p*-value < 0.001) suggests a strong relationship between the PMSSQ surface and its ability to adsorb chemicals based on physicochemical properties. This relationship could also be used to estimate the porewater concentration for PCB and other hydrophobic compounds based on the K_ow_ and concentration of chemicals adsorbed by the NPO film. Interestingly, the x-intercept of this relationship is around Kow 4, which would suggest very little binding of those compounds without additional surface modification of the NPO film.

## 4. Conclusions

This study demonstrates the silica nanoparticle platform for use as a passive sampler or potentially to pre-concentrate a target chemical as part of an active sensor system. The high surface area of this platform and simple silica foundation make this platform amenable to surface modification using a functional group or other binding site designed to target a specific chemical. The PMSSQ used to demonstrate this platform was intended to adsorb hydrophobic chemicals such as PCB. However, other surface modifications could be made to the surface of the nanoparticles to add different functional groups or receptors that could preferentially bind specific ligands or chemicals of interest. For example, there is a need for samplers or sensors that can measure the flux of metals, such as various species of mercury, from sediment systems. In this case, modification of the silica with a thiol-containing functional group would make this a system for metals. Other functional groups could include specific proteins such as antibodies to serve as a specific ligand targeting a group of chemicals. Other considerations for surface modifications must balance the properties such as hydrophobicity, robust physical surface, and minimal fouling that we demonstrated here for use in water and sediment. A challenge for this application is that it takes a long amount of time for equilibration, whereas many sensor technologies aim to detect chemicals in short amounts of time. Furthermore, the application of this platform for pre-concentration in sensor applications or for passive sampling would require a thorough characterization regarding its stability in the sample media, sensitivity, repeatability, potential to foul, and sorption kinetics and characteristics for the specific target chemical.

## Figures and Tables

**Table 1:** Optimization of nanoporous organosilicate (NPO) film curing temperature on polychlorinated biphenyl (PCB) adsorption on a total extracted and sampler volume-normalized basis over a 7-day period. Values represent an average of 3 replicate NPO films and standard deviation. Results are shown as both the total mass of PCB 153 and the mass of PCB 153 extracted normalized to sampler volume.

Curing Temperature (°C)	Surface Area (mm^2^)	Thickness (mm)	Volume (mm^3^)	PCB 153 (ng)	ng PCB 153/mm^3^ Sampler Volume
250	565 (101)	0.00176	0.994 (0.178)	8.2 (1.6)	8.5 (2.6)
350	477 (31)	0.00161	0.766 (0.050)	9.2 (3.5)	12.0 (4.2)
450	495 (45)	0.00137	0.678 (0.062)	10.0 (3.4)	14.9 (5.1)
550	469 (61)	0.000641	0.300 (0.039)	6.4 (2.0)	22.1 (8.8)

## Data Availability

All data reported here are available online [40], per the US Geological Survey Data Management Policy, at https://doi.org/10.5066/F7251H4B.
